# Optical fiber speckle spectrometer based on reversed-lens smartphone microscope

**DOI:** 10.1038/s41598-023-39778-z

**Published:** 2023-08-10

**Authors:** Henry Tan, Bingxi Li, Kenneth B. Crozier

**Affiliations:** 1 School of Physics, University of Melbourne, Parkville, VIC 3010 Australia; 2 ARC Centre of Excellence for Transformative Meta-Optical Systems (TMOS), University of Melbourne, Parkville, VIC 3010 Australia; 3 Department of Electrical and Electronic Engineering, University of Melbourne, Parkville, VIC 3010 Australia

**Keywords:** Optical spectroscopy, Applied optics

## Abstract

Smartphones are a potentially powerful platform for scientific instruments. Here, we demonstrate speckle spectroscopy with smartphone-level hardware. This technique promises greater performance thresholds than traditional diffraction gratings. Light is injected into an optical fiber and the emergent speckle patterns are imaged by a reversed-lens smartphone camera. The smartphone then uses an algorithm, running on a mobile computing app, to determine, in less than one second, the (hitherto unknown) input spectrum. We reconstruct a variety of visible-wavelength (470–670 nm) single and multi-peaked spectra using a tunable source. The latter also include a metameric pair, i.e., two spectra that are different, yet represent colors that are indistinguishable to the human eye.

## Introduction

There has recently been interest concerning the use of smartphones as platforms to realize low-cost and portable scientific instruments for applications that include mobile chemical analysis and point-of-care medical diagnostics^1^. Some notable developments have included microscopes for medical sample identification^2^, intensity fluorimeters for measuring water pH^3,4^ and grating spectrometers for biological assay examination^5^ and food testing^6,7^. In all cases, the scientific functionality is built into a module into which the smartphone is placed, and the design goal is to make the platform as small as possible.

Recent smartphone spectrometers use diffractive gratings/optics (e.g., DVD gratings^8^, blazed gratings^9^, G-Fresnel device^10^) to separate wavelengths by angle^11^. This changes where upon the camera sensor the light is detected. Diffractive spectrometer configurations do not require moving parts and are straightforward to keep calibrated. The linear spectral-to-spatial mapping can be determined by measuring a known spectral source. Smartphone grating spectrometers can disperse incoming light via reflection or transmission grating configurations. Transmission grating smartphone spectrometers usually have simpler construction, with the example of the G-Fresnel smartphone spectrometer achieving variable spectral resolutions of 1.3 to 5 nm in the visible spectrum. A reflection-grating configuration encourages longer effective path lengths because it folds over the optical path, with an example incorporating a fiber endoscope achieving a spectral resolution of 2 nm across the visible spectrum^12^.

The spectral resolution of grating spectrometers scales with the size of the grating and optical path length. But doing so increases the complexity and overall device size. Moreover, pushing resolutions higher would necessitate an order-of-magnitude increase in optical path length or grating size which is not compatible with the smartphone platform. Hence this motivates the exploration of smartphone-based spectrometers that use approaches other than gratings to enable high-resolution spectroscopy in small device footprints.

One interesting spectroscopic technique that could be possibly performed on a smartphone platform is speckle spectroscopy. In these systems, the input signal (whose spectrum is to be determined) is launched into a scattering element for which each spectral component produces a unique speckle pattern. The superposition of speckle patterns present then infers the original input spectrum. Scattering elements that have been employed in these systems include photonic crystals^13–15^, etched silicon waveguides^16,17^, multi-mode fibers (MMF), other fiber variants^18–22^ and integrating spheres^23,24^.

Some examples of what has been achieved are as follows. By imaging the speckle patterns produced at the end of a 100 m length of MMF and using a transmission matrix to model the patterns, Redding et al*.*^19^ demonstrated spectroscopy at $\lambda \sim 1500$ nm with a resolution of 1 pm over a 100 pm range. This length of fiber is contained in an ~ 8 cm diameter spool. In the same study, it was also demonstrated that a 4 cm length of MMF could be used for visible-wavelength spectroscopy covering the range 450–700 nm with a resolution of 1 nm. Significantly higher resolutions have also been shown possible. Using an integrating sphere, Metzger et al. applied principal component analysis to demonstrate a wavemeter at $\lambda \sim 780$ nm that is able to resolve narrow linewidth spectra spaced by 0.3 fm^23^. Utilization of multivariate modelling such as deep learning^24^, principal component analysis^25^, or Poincare descriptors^26^ has helped speckle wavemeters resolve narrower wavelength shifts, approaching attometer resolution in the case of Bruce et al^25^. These techniques have pre-dominantly been used to maximally characterize the decorrelations in the speckle patterns, thereby achieving highly sensitive wavemeters. Recently however, Bruce et al*.*^27^ used principal component analysis (PCA) with a unique calibration procedure to simultaneously retrieve two modulated laser lines that are on average separated by 22 fm. Each channel is resolved to within 0.2 fm accuracy. The calibration consisted of speckle patterns generated by simultaneous spectral channels that were independently wavelength modulated at different frequencies. Principal component analysis on the time-series of speckle patterns then revealed a one-to-one correspondence between principal components (PC) and individual channels, identified by modulation frequency. Parametrization of the monotonic relationship between PC and wavelength enabled each channel to be simultaneously and independently retrieved. It was nonetheless still apparent that there is a trade-off between spectral resolution and signal bandwidth retrievable.

Various multiplexing techniques have been explored to try to circumvent this resolution-bandwidth trade-off. Liew et al.^22^ worked around the bandwidth limitation of a single multimode fiber by incorporating a wavelength-division multiplexer and a 1-to-7 fan-out fiber bundle to distribute five spectral bands 20 nm wide into five different multimode fibers. They demonstrate speckle spectroscopy between 1520 and 1620 nm with 0.03 nm resolution. Another used space-division multiplexing whereby an optical switch and a 7-core fiber bundle coupled into a sole multimode fiber and is able to reconstruct spectra between 1520 and 1550 nm with 0.02 nm resolution^20^. The use of fiber bundles has also inspired other functionalities. Larger fiber bundles with thousands of fiber cores were used by French et al*.*^28^ to demonstrate hyperspectral imaging. The speckle patterns of each core carry the intensity and spectrum of a pixel in the images. Kürüm et al*.*^29^ later expanded upon this hyperspectral imaging concept with compressive-sensing and deep-learning to help speed up retrieval of specific wavelengths from hyperspectral images (e.g., smiley faces, rainbows, butterflies etc*.*) generated by a spatial light modulator.

There is a growing trend of miniaturizing optics to enable novel sensing applications. In the above-cited works, however, speckle experiments have prioritized pushing the frontiers of performance. This is achieved using complicated, expensive, and bulky optical and electronic systems. But this focus has left the popular suggestion of exploiting the technique for smaller spectrometers generally unanswered. A compact speckle spectrometer would need to substitute costly and bulky hardware with smaller and cheaper componentry. Recently, Malone et al. demonstrated a simple speckle spectrometer that uses Scotch tape as the scattering element^30^. They reconstructed narrowband and broadband spectra with 2 nm resolution in the 785 nm to 870 nm range. But the speckle patterns were imaged using a scientific-grade camera. These setups, sometimes augmented with high-magnification objectives, are a high-performance solution but are costly and large. An alternative approach is to perform the imaging with a smartphone. The computing performance and camera systems in smartphones have been leveraged to demonstrate portable scientific sensing systems such as microscopes^3^ and spectrometers^31^.

Here, we demonstrate that a smartphone can be used to both image speckle patterns emergent from optical fibers and as a computational platform to reconstruct the input spectrum. An inexpensive reversed lens is attached to the smartphone to enable it to image the speckle patterns. This imaging system reduces the number of expensive optical elements required. These comprise the smartphone, a reversed-lens camera module, the fiber, and a fiber coupler. MMF is chosen as the scattering medium because of its ability to be coiled into a small volume and wide availability. We demonstrate that it is nonetheless capable of reconstructing various spectra (in the wavelength range 470–670 nm) and of resolving spectral features to the nearest 2 nm. The results shed hopeful light on the prospects of implementing speckle spectroscopy with lower-end hardware. Some earlier aspects of this work has been shown in a brief conference proceeding^32^.

### Using a smartphone to analyse speckle patterns

**Figure 1:**
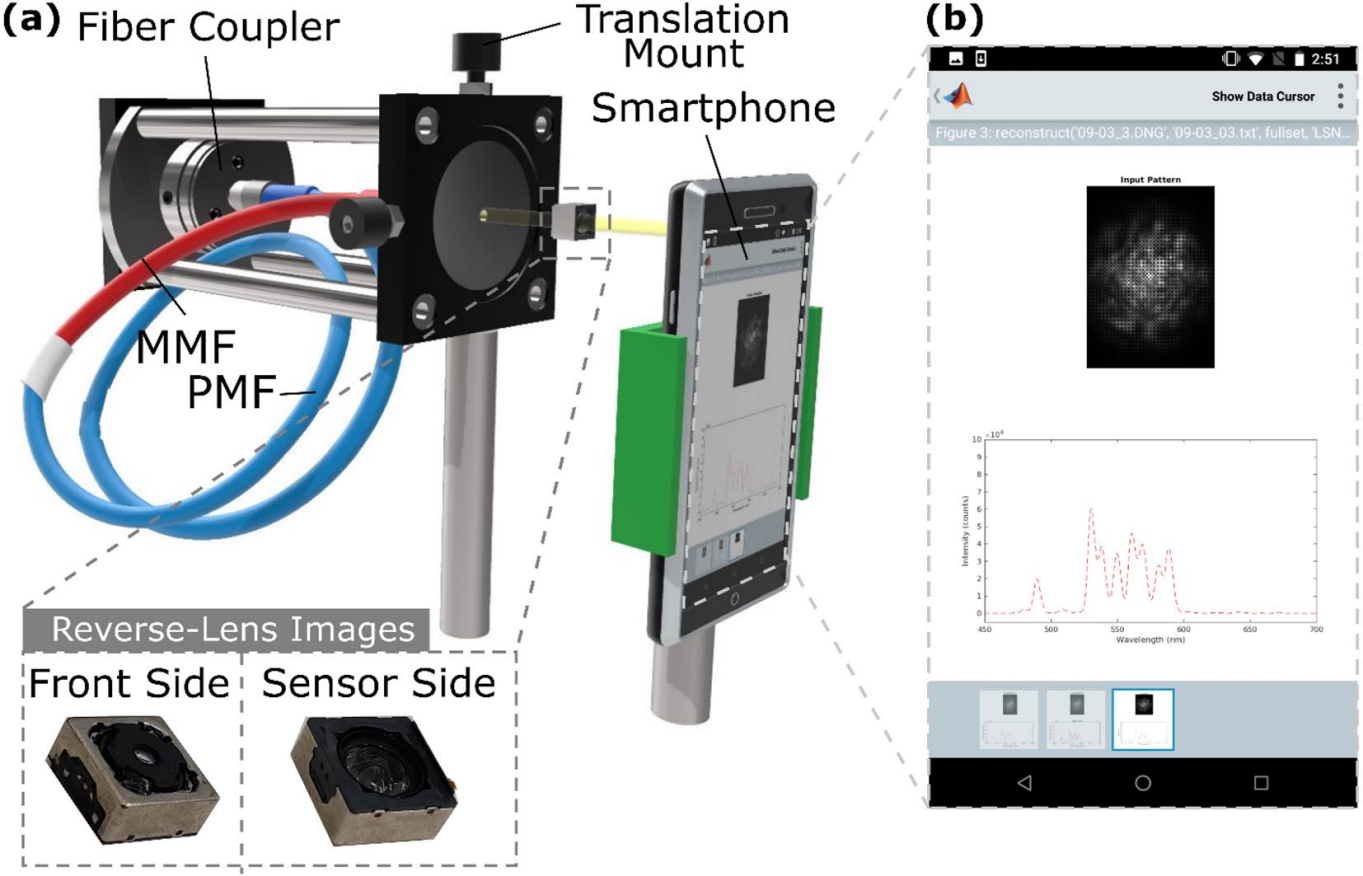
(**a**) Schematic of experimental setup. (Inset) Close-up photo of camera module with sensor removed, i.e., reversed-lens. (**b**) Screenshot of mobile interface showing input speckle pattern and reconstructed spectrum.

A schematic of the smartphone spectrometer of the experimental setup is shown (Fig. 1a). The sample light is coupled into the optical fiber by a fiber coupler. The fiber consists of polarization-maintaining single-mode fiber (PMF, Thorlabs PM460-HP, 1 m long, depicted in blue cladding), spliced to a multi-mode fiber (MMF, Thorlabs FG105UCA, 5 cm long, core-diameter = 105 µm, NA = 0.22, depicted in red cladding). The polarization-maintaining single-mode fiber splicing provides a consistent coupling interface with the multi-mode fiber to ensure predictable speckle generation. In ordinary single-mode fiber, optical power randomly couples between orthogonal polarizations. This is problematic as orthogonal polarizations will produce different speckle patterns from the MMF. Polarization-maintaining fiber maintains the linear polarization of light propagating along slow/fast axes of the fiber. A linear polarizer would ordinarily be used to ensure correct launching conditions into the optical fiber. In this work, the experimental realization of the speckle smartphone spectrometer is limited to an optics bench demonstration. Here a supercontinuum white light source (SuperK Compact) and tunable filter (SuperK Multi-line Select) is used to both calibrate the spectrometer and generate test spectra. This tunable source combination produces a linearly polarized output and thus a polarizer was not used. The output impinges upon a 50:50 beam splitter where the reflected light is diverted to a reference spectrometer (OceanOptics QE-Pro, integration time = 25 µs) and the transmitted light can pass into the speckle spectrometer. This configuration allows the calibrated speckle spectrometer to be characterized, using the source to generate single or multi-peaked spectra that are recorded by the reference spectrometer for comparison to the smartphone spectrometer’s reconstruction. Also, the smartphone’s screen can be used to display useful information such as the input image and the reconstructed spectrum (Fig. 1b).

**Figure 2:**
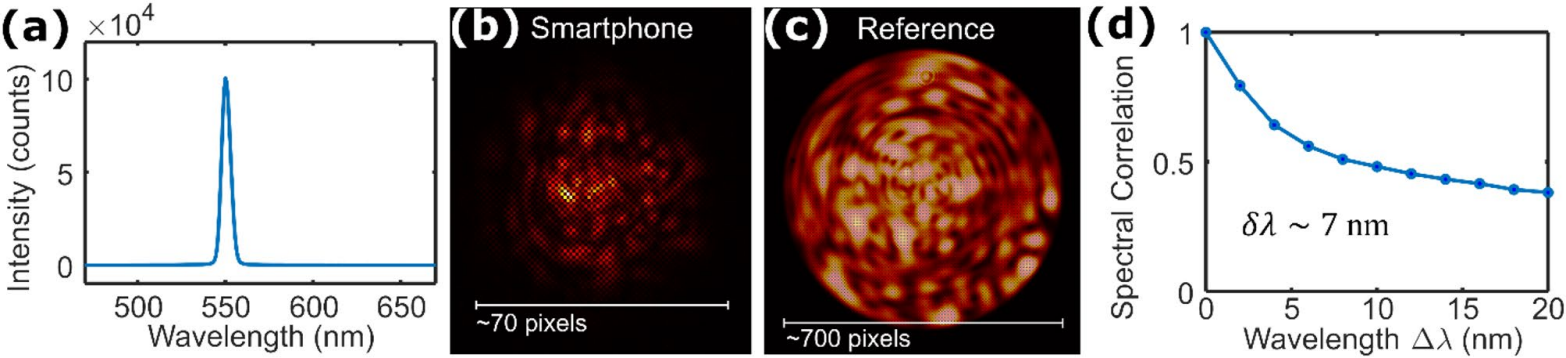
(**a**) Example of source spectrum (here centered at 550 nm) used in calibration step, as measured by a reference spectrometer. (**b**) Representative speckle pattern recorded by smartphone microscope (significant image crop, false color). (**c**) High-quality, reference image of speckle pattern (40× magnification, false color). The speckle pattern covers approximately 700 × 700 pixels. (d) Measured spectral correlation.

A reversed smartphone camera lens (Fig. 1a inset) is attached to the smartphone (LG Nexus 5x) to enable it to satisfactorily image the speckle patterns emergent from the optical fiber. The additional lens module, identical to the one the smartphone uses, is placed in front, but in the reversed orientation, of the smartphone camera (Sony IMX377EQH5, 3000 × 4000 pixels). This reversed lens converts the smartphone into a microscope with unity magnification^2^. When a narrowband signal centered at 550 nm (Fig. 2a) is launched into the system, the speckle pattern captured has a width of ~ 70 pixels across (Fig. 2b). The dark gridded artefact present in the image is due to the red–green–green–blue (RGGB) color filter array atop the sensor. Normally this filter array, in tandem with demosaicing, is responsible for generating color images, but here we opt to leave the image in its raw form to preserve detail and contrast helpful for speckle reconstruction. The quality of the smartphone is better understood when compared to a higher-quality reference image of the speckle patterns typically produced by the fiber. The reference image shown in Fig. 2c is taken with a scientific color camera (Point Grey Flea 3, 960 × 1280 pixels) with 40 × magnification. It is also generated by light with a wavelength of 550 nm. The speckle patterns are significantly larger, measuring ~ 700 pixels across, and show the speckle intricacy lost with the smartphone system. Yet despite the image degradation, the smartphone images are still surprisingly viable for speckle reconstruction.

The process by which the speckle spectrometer is calibrated is as follows. When light is launched into the fiber, each spectral component excites a combination of guided modes^33^ that interfere to produce the speckle pattern imaged by the smartphone. Although there has been work into theoretically modelling certain propagation modes in MMF^34^, it is still not possible to calculate from first principles the exact speckle pattern that would be produced by a given monochromatic signal, as such patterns are sensitive to experiment-specific details, e.g., geometric configuration of fiber^35^. Thus, we must instead record it experimentally. This is performed using the tunable filter to generate single-peaked spectra over the wavelength range 470–670 nm in steps of 2 nm. The maximum tuning range of the system is 450 nm to 670 nm, but the optical power below 470 nm is too low for the smartphone camera. The speckle patterns and spectra are recorded at each step by the smartphone camera and reference spectrometer, respectively. This creates a calibration library of 101 pairs of calibration patterns and spectra which is then used to perform spectral reconstruction.

### Spectrum calculated from speckle pattern reconstruction

Speckle patterns are generated in a linear and deterministic manner provided that the scattering media (e.g., optical fiber) remains undisturbed. Each monochromatic wavelength will continue to excite the same combinations of modes in the fiber to produce a unique speckle pattern. Thus, the resultant speckle pattern of any broadband input spectrum is the superposition of speckle patterns generated by each wavelength component present. The transmission matrix method models speckle patterns $I$ as a linear combination ${S}_{I}$ of speckle patterns stored in an eponymous transmission matrix $T$ such that: $I=T\cdot {S}_{I}$. The speckle pattern images are converted to a vector by sampling pixels within the illuminated area of each pattern, i.e., this vector represents a smaller portion of the pixels in each picture captured by the image sensor. This vector is akin to a barcode preserving the uniqueness of each speckle pattern’s fingerprint. In theory, once the transmission matrix is calibrated, solving the inverse problem will retrieve the spectrum of any speckle pattern $I$.

The transmission matrix method works best when decomposing speckle patterns into a maximally uncorrelated basis. The best spectral resolution achievable with this model is then related to the wavelength separation required for speckle patterns to be sufficiently decorrelated. This can be understood as the average wavelength shift $\delta \lambda$ that can cause two modes to experience a $\pi$-phase difference, i.e., shifting from constructive to destructive interference and vice versa. In MMF, this is theoretically approximated in^18^ as $\delta \lambda \sim {\lambda }^{2}/[nL{\left(NA\right)}^{2}]$ where $\lambda$ is the wavelength of the signal, $n$ is the effective refractive index of the MMF, $L$ is the length of MMF and $NA$ is the numerical aperture. So, for our MMF with $n=1.45$, $NA=0.22$ and $L=5$ cm, we have an upper estimate threshold of $\delta \lambda \sim 0.1$ nm at $\lambda =550$ nm. In practice though, the spectral resolution will also depend upon the complexity of the speckle pattern generated (i.e., number of modes excited^36^), image quality, reconstruction method employed, and the linewidth of the calibration source used.

The experimentally measured speckle intensity decorrelation is quantified by the following spectral correlation function: $C(\Delta \lambda ,x)=\langle I\left(\lambda ,x\right)I\left(\lambda +\Delta \lambda ,x\right)\rangle /\left[\langle I\left(\lambda , x\right)\rangle \langle I\left(\lambda +\Delta \lambda ,x\right)\rangle \right]-1$, where $I(\lambda ,x)$ is the intensity at a position $x$ of a speckle pattern generated by wavelength $\lambda$ and $\langle \dots \rangle$ is the average over all wavelengths. This quantity is averaged over all $x$ to yield $C(\Delta \lambda )$, and for our system is shown in Fig. 2d. The spectral correlation width $\delta \lambda$ is then defined such that $C\left(\delta \lambda \right)=C(0)/2$, which is ~ 7 nm for our system. The tunable source that we use for the experiments has a single wavelength channel spectral width of ~ 5.5 nm (averaged over 470 nm to 670 nm). The spectral correlation width estimates the narrowest separation between two adjacent wavelengths that can be distinguished by the spectrometer. We nonetheless use a finer step-size in the calibration because the increased sampling can help identify sparse spectral features more precisely without the additional correlated speckle patterns contributing to excess reconstruction noise.

The nature of the transmission matrix $T$ renders its direct inversion an inaccurate means of estimating ${S}_{I}$^18^. This is because it contains experimental noise and collects many near-zero values from image dark zones^37^. In addition, it is in general not square, i.e., the number of pixels sampled from each speckle pattern vastly outnumbers the number of speckle patterns in the calibration set. We instead must find an approximation ${S}_{I}^{\prime}$ that best reproduces the observed speckle pattern. Once found, the unknown spectrum $S^{\prime}$ can be reconstructed as a linear combination of the spectra of the speckle patterns found within, i.e., ${S}^{\prime}=A\cdot {S}_{I}^{\prime}$ where, analogous to the transmission matrix for speckles, the spectral conversion matrix $A$ contains the spectra of the calibration patterns along its columns^38^.

The inverse problem outlined above is fundamental to many spectrometers that computationally reconstruct the input spectrum^39–43^ rather than doing direct read-out. In our case, solving for ${S}_{I}^{\prime}$ is performed using the least-squares non-negative solver, lsqnonneg function, of the software package MATLAB (MathWorks, Massachusetts, USA). This uses an algorithm described in^44^ which does not employ probabilistic methods (like simulated annealing) or require experimentally- and computationally expensive training (like deep-learning). This function takes the measured speckle pattern $I^{\prime}$ as input and estimates the value of ${S}_{I}$ by minimizing the cost functions ${\left|I-{I}^{\mathrm{^{\prime}}}\right|}_{2}={\left|I-T\cdot {S}_{I}^{\mathrm{^{\prime}}}\right|}_{2}$ and ${\left|{S}_{I}^{\mathrm{^{\prime}}}\right|}_{2}$, subject to the non-negative constraint ${S}_{I}^{\mathrm{^{\prime}}}>0$. Here ${\left|...\right|}_{2}$ denotes the L2-norm and ${S}_{I}^{\prime}>0$ means that every element in ${S}_{I}^{\prime}$ is non-negative. The minimization of ${\left|{S}_{i}^{\prime}\right|}_{2}$, which is done in tandem with the minimization of ${\left|I-T\cdot {S}_{I}^{\prime}\right|}_{2}$, results in smaller-valued solutions as larger discrepancies are penalized more harshly. The non-negative constraint follows from non-physicality of negative values in the speckle patterns and spectra. The mean spectral error^18^, a measure of how well the spectral reconstruction $S^{\prime}$ matches the target spectrum $S$, is defined to be the standard deviation of the difference between the two: $MSE=\sqrt{\langle {\left(S-{S}^{\prime}\right)}^{2}\rangle }/\langle S\rangle$.

MATLAB mobile is an application available on Android™ or iOS devices that conveniently lets users run MATLAB scripts from their smartphones. Currently, script execution is facilitated through MATLAB’s MathWorks cloud computing service only. Also, scripts can only access data stored online on MATLAB Drive. The calibration library of speckle patterns and spectra are uploaded to the drive, and the transmission and spectral conversion matrices are computed. These are saved on the drive for quick reference. Thereafter, the spectrum of a speckle pattern imaged by the smartphone can be found by running the reconstruction algorithm script via the application. This cloud-based service returns the results to the smartphone in less than a second. This compares to only a few seconds when the script is run locally on a dual-core tablet PC (Intel^®^ Core™ i5-4300U Processor). It is unlikely such algorithms will take much longer if run locally on modern smartphones.

## Results

**Figure 3:**
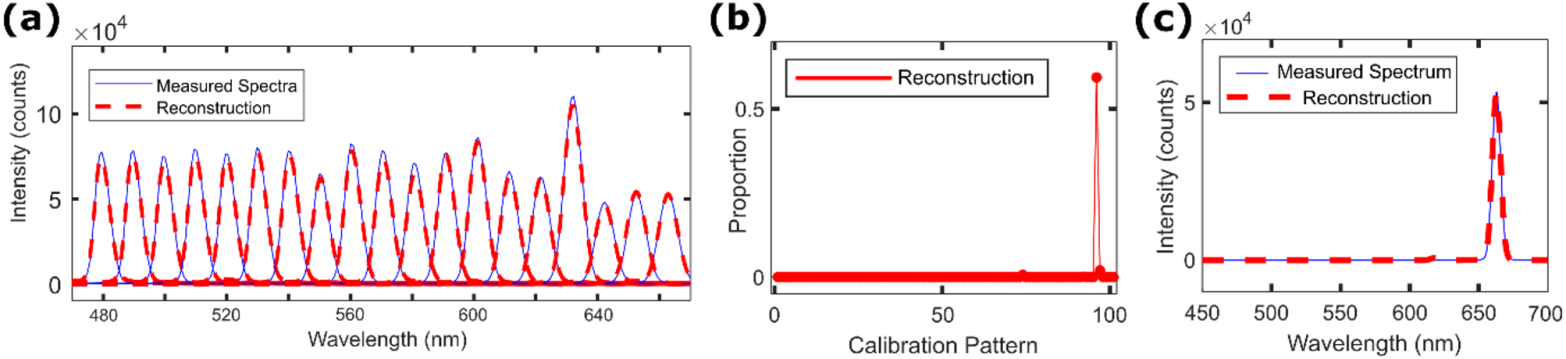
(**a**) Spectral reconstruction of 19 different single-peak spectra. The average mean spectral error (MSE) of the group is 0.21. (**b**) Linear combination of speckle patterns ${S}_{I}^{\prime}$ of the best performing test. (**c**) Corresponding spectral reconstruction, MSE = 0.07.

We first test our smartphone spectrometer by reconstructing a series of single-peaked spectra like those used for calibration. The reconstructions produced by the smartphone spectrometer (dashed red curves, Fig. 3a) are in very good agreement with those measured by the reference spectrometer (blue curves, Fig. 3a). The mean spectral error for the 19 reconstructions is $\langle MSE\rangle =0.21$. In Figs. 3b and c, we single out the best-performing test. The speckle reconstruction ${S}_{I}^{\prime}$ (Fig. 3b) displays minimal crosstalk and its corresponding spectral reconstruction $S^{\prime}$ (Fig. 3c) has $MSE=0.07$. This indicates that the speckle reconstruction algorithm can correctly identify a single speckle pattern to its nearest 2 nm neighbour. We thus identify spectral features to the nearest 2 nm, though expect that it could be possible to surpass this (and achieve resolution closer to the theoretical estimate) using a calibration source with narrower linewidths.

**Figure 4:**
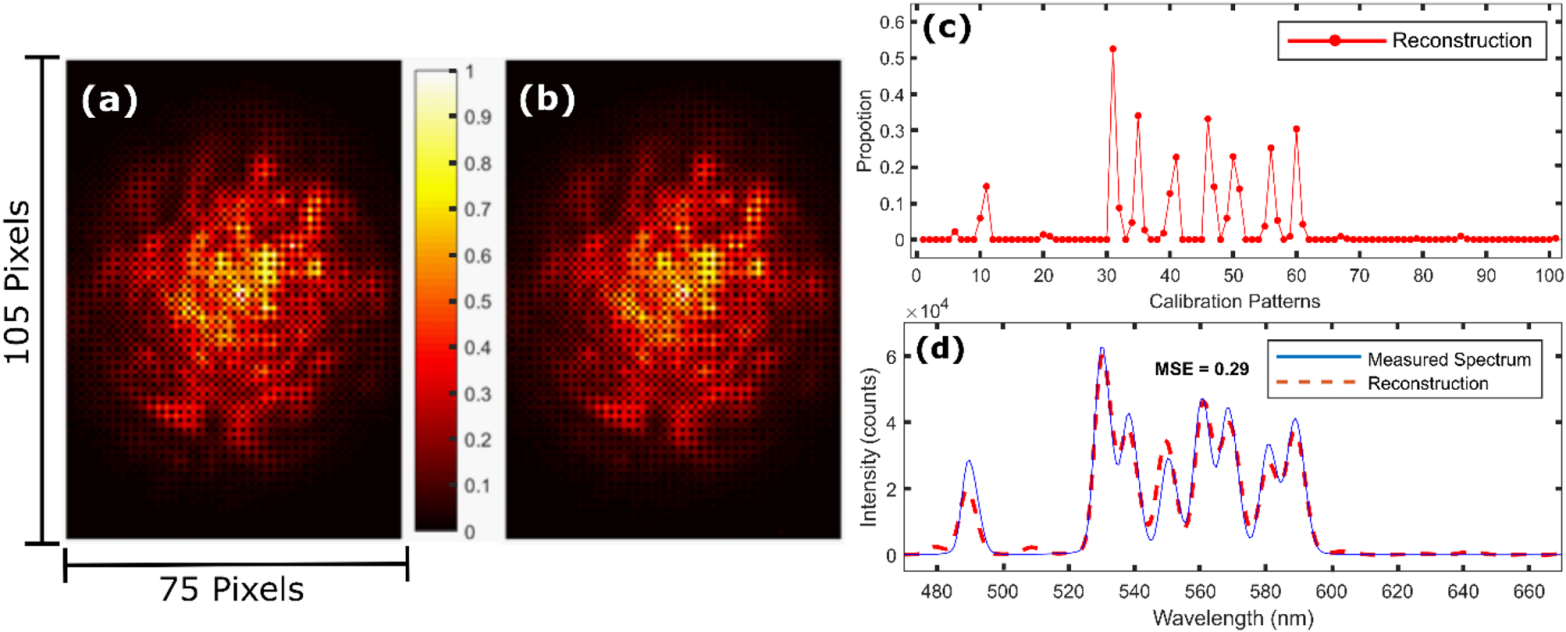
(**a**) False-color image of a speckle pattern. (**b**) Reconstructed speckle image based upon the ${S}_{I}^{\prime}$ solution shown in (**c**). (**d**) The resultant spectral reconstruction, MSE = 0.29.

We next test our speckle spectrometer with a broader input spectrum, again generated by the tunable filter (SuperK SELECT) supercontinuum (SuperK Compact) combination. The false-color image of the speckle pattern captured by the smartphone is shown as Fig. 4a. This is provided to the reconstruction algorithm, which then estimates the input spectrum as before. The reconstructed speckle image, the ${S}_{I}^{\prime}$ reconstruction and the reconstructed spectrum are shown as Fig. 4b–d, respectively. The reconstructed speckle image (Fig. 4b) is in close resemblance of the measured speckle image (Fig. 4a). As such, the reconstructed spectrum (Fig. 4d) is in good agreement with the spectrum measured by the reference spectrometer, with MSE = 0.29. The good agreement observed, even for such a broad input spectrum, arises from the fact that the spectrum can be well-expressed as sparse in the reconstruction domain^45^ (Fig. 4c).

**Figure 5:**
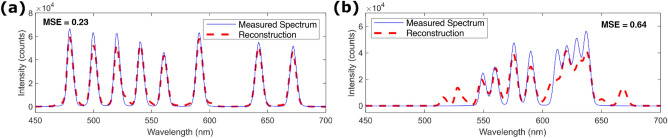
(**a**) Reconstruction of a sparse spectrum in comparison to a spectrum with denser features in (**b**).

Sparsity here refers to spectra that have spectral features sufficiently spaced such that the speckle patterns of each component are minimally correlated with one another. In this condition, the recovery of the speckle pattern decomposition is maximized, leading to a more accurate reconstructed spectrum (Fig. 5a). Our choice of a calibration with wavelength separations smaller than the average full-width half-maximum of the linewidth means that adjacent channels are not truly independent of each other. In densely packed spectra, the algorithm has some difficulty determining the accurate proportion of each speckle pattern present (Fig. 5b). Here the upward trend of the broad signal in the 600 nm to 650 nm region is followed, but the four defined spectral peaks are largely missed, with additional spurious out-of-band contributions. The compromise in using a sparser calibration basis to minimize noisy reconstruction of broader spectra is that it comes at the cost of worse precision when identifying isolated spectral features.

**Figure 6:**
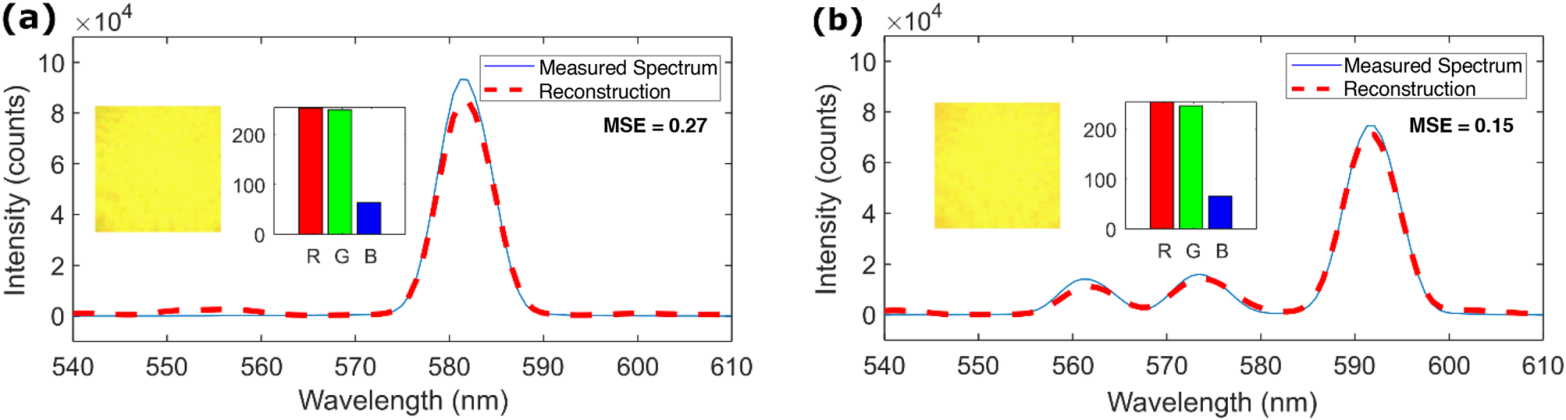
(**a**) and (**b**) are the reconstruction of a pair of metameric spectra, MSE = 0.27 and 0.15 respectively. (Insets) Color image of beam center and accompanying mean red–green–blue values.

Lastly, we reconstruct a pair of metameric spectra, with the results shown in Fig. 6. Metamerism is the phenomenon where different spectral distributions appear to be the same color to the human eye or color cameras^14^. Two test spectra are generated by the tunable filter/supercontinuum source combination. These are chosen to have nearly identical colors (i.e., red/green/blue values) but have different spectra. To quantify the colors of the beams, we image each beam in a dark environment by a separate camera (Point Grey Flea 3) placed directly in front of the reference spectrometer (Fig. 6, inset). This is later removed to measure the reference spectrum of the signal. The RGB values are shown on a scale between 0 and 255 because of its ubiquity in popular computer graphics editor software. The mean red–green–blue (RGB) values of the beam centers are very similar, with the first beam (Fig. 6a) having [R, G, B] = [253.9, 250.1, 62.7] and the second beam (Fig. 6b) having [R, G, B] = [254.9, 247.7, 64.7]. The differences in the mean RGB values are all less than 2.4 units, with green values exhibiting the largest percentage difference of 3.2%. Small dark streaks present in the image are likely from the imperfections on the camera’s glass cover. Whilst it is difficult to distinguish between the beams based on their RGB values, the smartphone spectrometer can do so. The first beam (Fig. 6a) has a spectrum with a prominent peak centered at around 582 nm, while the second beam (Fig. 6b) has a main peak at around 592 nm and two smaller peaks at 562 and 574 nm. The smartphone spectrometer can make the distinction between the metameric test spectra because the reconstruction makes use of the spatial distribution of the light exiting the fiber (i.e., speckle patterns) rather than just its RGB color values.

## Discussion

Here we demonstrate an unusual spectroscopy technique unseen before in the realm of smartphone-sensing. Speckle spectroscopy promises significantly higher resolution thresholds than that currently achieved by grating-based smartphone spectrometers. Pursuing higher resolutions with grating-based designs would require an order of magnitude increase to the optical path length or grating size that may not fit the small smartphone form-factor. However, using smartphone hardware for speckle spectroscopy is not without its own disadvantages and uncertainties.

The spectral resolution is not definitively pushed in this iteration because of the tunable source used and the quality of the smartphone pictures. Unsurprisingly, the smartphone images (Fig. 2b) in comparison have worse contrast, lower brightness, have vignetting and are blurrier than those taken with a scientific imaging setup (Fig. 2c). But the smartphone appears to be able to reconstruct spectral features approaching the $\delta \lambda \sim 7$ nm limit. Thus, it may be possible that the system in its current form could reconstruct spectra more accurately if calibrated with a narrower linewidth source. Still, other smartphone camera systems may respond better to the reverse-lens imaging paradigm. Alternatively, a custom-housing to hold the smartphone and reverse-lens may facilitate superior optical alignment.

Currently, the low efficiency of coupling free-space incoherent light into a single-mode fiber renders at-a-distance stand-off spectroscopy improbable. One could add additional lenses to improve light collection, but the fiber’s small numerical aperture cannot be easily overcome. Instead of measuring far away sources, one may opt to perform reflection or transmission mode spectroscopy of samples using external sources (e.g., smartphone’s flash) that can provide sufficient illumination. One idea similar to^12^ is to incorporate an endoscopic fiber bundle to probe samples and couple light into an internal optical fiber responsible for speckle generation.

Stability is a real-world concern for all speckle spectrometers. Mechanical shocks and vibrations that bend the fiber out of its initial position will irreversibly change the speckle patterns generated and thus require re-calibration. Shock resistance may be improved by setting the entire fiber and optics in epoxy resin. This would limit serviceability but likely reduce the probability of catastrophic speckle decorrelation. The speckle patterns may also decorrelate because of temperature fluctuations changing the fiber’s refractive index and length via the thermo-optic effect and thermal expansion. But fortunately, in^19^, the authors found that for small temperature changes (e.g., $\sim$ 10 degrees Celsius for 4 cm MMF), the phase delay introduced by both effects is approximately the same across all modes in the MMF and thus can be accounted for by applying a wavelength shift. In effect, the speckle patterns are collectively red-shifted proportional to temperature increases and vice versa. In the future, the fiber’s temperature could be monitored and potentially controlled with a thermoelectric heater/cooler. But further investigation into such implementation will be needed. For example, it is unclear if there are temperature hysteretic effects may affect the fiber’s return to its initial state. Alternatively, different scattering medium could be utilized such as disordered photonic chips or etched silicon waveguides. These offer more mechanical robustness over flexible fiber. These may be more easily incorporated with smartphone components for ultra-small spectrometers.

## Conclusion

In summary, we have shown that a smartphone with a reversed lens can image speckle patterns at the end of multimode fiber and function as a speckle spectrometer. Although the magnification is smaller than what would be achievable with a microscope objective, the smartphone microscope manages to capture enough detail to render good results (Figs. 3, 4, 5 and 6). We show it can provide broadband operation (470–670 nm) and resolve features to the nearest 2 nm. The spectrometer functionality requires few parts; aside from the smartphone, comprising of only a fiber coupler, an optical fiber, and a reversed lens. This should help it to fit inside a custom module no larger than a typical smartphone battery case. Although this current implementation of speckle spectroscopy does not exceed the current state-of-the-art grating spectrometers, it is a first look at an alternative technique that has not been explored on the platform. This demonstrates the potential flexibility of a smartphone-based system that uses unusual, or non-traditional, methods of sensing. Although the initial calibration is more labor-intensive and requires specialized equipment, these trade-offs may be workable if it can unlock performance that exceeds that attainable from gratings spectrometers of comparable size.

## Data Availability

The datasets used and/or analyzed during the current study is available from corresponding author upon reasonable request.
